# Supplementary material for: SARS-CoV-2 pneumonia follow-up and long COVID in primary care: A retrospective observational study in Madrid city
Source: PLoS One. 2021 Sep 22;16(9):e0257604. doi: 10.1371/journal.pone.0257604 (PMC8457448; doi:10.1371/journal.pone.0257604)
Supplement: S1 File — (DOCX) [file pone.0257604.s001.docx]

Supplementary file 1: Clinical characteristics of patients with SARS-CoV-2 pneumonia follow-up in primary care during 6 month, stratified by age groups (2020).

|  | **<50 (n:48)** | **50-75 (n: 77)** | **>75 (n: 30)** | ***p*-value** |
| --- | --- | --- | --- | --- |
| **Sociodemographic** |  |  |  |  |
| Age (years) ^#^ | 39.0 (8.3) | 62.2 (6.5) | 81.9 (5.8) |  |
| Sex (women) ^&^ | 25 (52) | 31 (40) | 24 (80) | <0.001 |
| Foreigner ^&^ | 17 (35) | 18 (23) | 1 (3) | 0.005 |
| **Comorbidities** |  |  |  |  |
| Overweight (BMI >25)^&^ | 16 (33) | 48 (62) | 19 (63) | 0.003 |
| Hypertension ^&^ | 6 (12) | 398 (49) | 27 (90) | <0.001 |
| Dyslipidaemia ^&^ | 11 (23) | 30 (39) | 19 (63) | 0.002 |
| Type II Diabetes ^&^ | 0 (0) | 19 (25) | 10 (33) | <0.001 |
| Asthma ^&^ | 4 (8) | 7 (9) | 9 (30) | 0.008 |
| Smoke habit ^&^ | 3 (6) | 9 (12) | 0 (0) | 0.064 |
| CKD ^&^ | 0 (0) | 3 (4) | 8 (27) | <0.001 |
| Cancer ^&^ | 0 (0) | 4 (5) | 6 (20) | 0.002 |
| COPD ^&^ | 1 (2) | 5 (6) | 2 (7) | 0.510 |
| Heart failure ^&^ | 0 (0) | 3 (4) | 4 (13) | 0.021 |
| **Symptoms until pneumonia diagnosis** |  |  |  |  |
| Fever (>37,5ºC)^&^ | 41 (85) | 69 (90) | 21 (70) | 0.041 |
| Cough ^&^ | 43 (90) | 65 (84) | 22 (73) | 0.160 |
| Dyspnoea ^&^ | 29 (60) | 46 (60) | 16 (53) | 0.800 |
| Myalgias ^&^ | 15 (31) | 28 (36) | 4 (13) | 0.066 |
| Asthenia ^&^ | 13 (27) | 17 (22) | 10 (33) | 0.480 |
| Headache ^&^ | 13 (27) | 17 (22) | 10 (33) | 0.480 |
| Chest pain ^&^ | 17 (35) | 7 (9) | 1 (3) | <0.001 |
| Dysgeusia ^&^ | 6 (12) | 2 (3) | 0 (0) | 0.019 |
| Anosmia ^&^ | 1 (2) | 1 (1) | 0 (0) | 0.730 |
| GI symptoms ^&^ | 23 (48) | 30 (39) | 14 (47) | 0.560 |
| Number of symptoms |  |  |  |  |
| 1- 3 ^&^ | 20 (42) | 38 (49) | 25 (83) | <0.001 |
| ≥ 4 ^&^ | 28 (58) | 38 (50) | 5 (17) |  |
| **Symptoms during follow up** |  |  |  |  |
| Fever (>37,5ºC) ^&^ | 3 (6) | 7 (9) | 1 (3) | 0.570 |
| Cough ^&^ | 24 (50) | 32 (42) | 9 (30) | 0.220 |
| Dyspnoea ^&^ | 20 (42) | 21 (27) | 7 (23) | 0.140 |
| Myalgias ^&^ | 9 (19) | 11 (14) | 2 (7) | 0.300 |
| Asthenia ^&^ | 12 (25) | 24 (31) | 5 (17) | 0.300 |
| Headache ^&^ | 10 (21) | 8 (10) | 0 (0) | 0.018 |
| Chest pain ^&^ | 11 (23) | 12 (16) | 2 (7) | 0.160 |
| Dysgeusia ^&^ | 0 (0) | 2 (3) | 0 (0) | 0.019 |
| Anosmia ^&^ | 2 (4) | 2 (3) | 0 (0) | 0.530 |
| GI symptoms ^&^ | 14 (29) | 20 (26) | 7 (23) | 0.840 |
| Number of symptoms |  |  |  |  |
| 1- 3 ^&^ | 25 (52) | 44 (57) | 18 (60) | 0.100 |
| ≥4 ^&^ | 16 (33) | 22 (29) | 3 (10) |  |
| **Chest X-ray at diagnosis*** |  |  |  |  |
| Bilateral pneumonia ^&^ | 27 (56) | 59 (77) | 24 (80) | 0.024 |
| **Blood test at diagnosis** |  |  |  |  |
| Lymphocytes (10E3/Âµ) ^$^ | 1250 (1000, 1750) | 1050 (800, 1400) | 1000 (700, 1500) | 0.021 |
| D-dimer (μg/L) ^$^ | 410 (240, 901.5) | 460 (270, 880) | 429 (320, 980) | 0.710 |
| Ferritin (μg/L) ^$^ | 474 (320, 1738) | 494.0 (203.0, 913.0) | 242 (176, 1215) | 0.590 |
| CRP (mg/L) ^$^ | 57.0 (12.8, 115) | 59.6 (30.0, 137.2) | 71.1 (41.8, 118) | 0.650 |
| **Chest- Xray follow-up (day)**** | 52.7 (26.5) | 54.4 (27.4) | 49.3 (38.6) | 0.750 |
| Normal X-ray ^&^ | 44 (92) | 45 (58) | 9 (30) | <0.001 |
| **Blood test follow-up (day)**** | 33.7 (16.2) | 34.0 (13.9) | 40.7 (15) | 0.120 |
| Lymphocytes (10E3/ÂµL) ^#^ | 2739.5 (798.2) | 2334.3 (816.6) | 2240 (1219.6) | 0.045 |
| D-dimer(μg/L) ^$^ | 290 (210, 670) | 430 (260, 640) | 1195 (744.5, 228) | <0.001 |
| CRP (mg/L)^$^ | 0.7 (0.3, 1.9) | 1.4 (0.3, 4.9) | 2.1 (0.3, 4.0) | 0.140 |
| Ferritin (μg/L) ^$^ | 95 (38, 266) | 199.5 (86, 429) | 55.5 (19, 93) | 0.033 |
| **Acute complications** |  |  |  |  |
| Hospital Admission ^&^ | 30 (62) | 64 (82) | 30 (100) | <0.001 |
| ICU admission ^&^ | 1 (2) | 3 (4) | 0 (0) | 0.500 |
| **Follow-up at practice** |  |  |  |  |
| Pneumonia onset (days) ^#^ | 8.1 (4.7) | 8.4 (3.9) | 7 (3.4) | 0.310 |
| GP´s follow up until recovery ^$^ | 53.0 (40.0, 71.5) | 54.0 (44.0, 91.0) | 63.0 (43.0, 92.0) | 0.550 |
| Phone calls (number) ^#^ | 12.6 (7.3) | 12.2 (6.4) | 11.5 (5.2) | 0.780 |
| **Long COVID complications** |  |  |  |  |
| Thromboembolism ^&^ | 3 (6) | 3 (4) | 0 (0) | 0.380 |
| Readmission ^&^ | 3 (6) | 4 (5) | 2 (7) | 0.950 |
| Home Oxygen Therapy ^&^ | 0 (0) | 3 (4) | 5 (17) | 0.004 |

**Legend**. &: number (%), #: mean (standard deviation), $: median (interquartile range), BMI (body mass index), COPD (chronic obstructive pulmonary disease), GI (gastrointestinal), ICU (intensive unit care); CRP (C reactive protein).
