# Supplementary material for: SARS-CoV-2 pneumonia follow-up and long COVID in primary care: A retrospective observational study in Madrid city
Source: PLoS One. 2021 Sep 22;16(9):e0257604. doi: 10.1371/journal.pone.0257604 (PMC8457448; doi:10.1371/journal.pone.0257604)
Supplement: S2 File — (DOCX) [file pone.0257604.s002.docx]

Supplementary file 2: Logistic regression analyses of the influence of suffering long COVID (>4 weeks symptoms) on patient´s characteristics and symptoms.

|  | **Model 1** | **Model 2** |
| --- | --- | --- |
|  | **OR (95% CI)** | **OR (95% CI)** |
| Sex (male) | 2.08 (1.005-4.305) | 2.47 (0.792-7.710) |
| Age | 0.98 (0.960-1.002) | 1.00 (0.970-1.040) |
| Cough´s duration |  | 1.13 (1.073-1.192) |
| Dyspnoea´s duration |  | 1.08 (1.043-1.135) |
| Asthenia´s duration |  | 1.11 (1.037-1.188) |

**Legend.** OR (odds ratio), CI (confidence interval).
