# Supplementary material for: SARS-CoV-2 pneumonia follow-up and long COVID in primary care: A retrospective observational study in Madrid city
Source: PLoS One. 2021 Sep 22;16(9):e0257604. doi: 10.1371/journal.pone.0257604 (PMC8457448; doi:10.1371/journal.pone.0257604)
Supplement: S3 File — (DOCX) [file pone.0257604.s003.docx]

Supplementary file 3: Differences between <12 weeks and ≥12 weeks of clinical course of SARS-CoV-2 pneumonia patients

|  | **≤ 12 weeks**  (n:142) | **> 12 weeks**  (n:13) | ***p*-value** |
| --- | --- | --- | --- |
| **Sociodemographic** |  |  |  |
| Age (years) ^#^ | 59.0 (16.9) | 56.5 (15.6) | 0.600 |
| Sex (women) ^&^ | 72 (50.7) | 8 (61.5) | 0.450 |
| Foreigner^&^ | 35 (24.6) | 1 (7.7) | 0.170 |
| **Comorbidities** |  |  |  |
| Overweight (BMI >25) ^&^ | 76 (53.5) | 7 (53.8) | 0.670 |
| Hypertension^&^ | 67 (47.2) | 4 (30.8) | 0.260 |
| Dyslipidaemia^&^ | 56 (39.4) | 4 (30.8) | 0.540 |
| Type II Diabetes^&^ | 26 (18.3) | 3 (23.1) | 0.670 |
| Asthma^&^ | 20 (14.1) | 0 (0.0) | 0.150 |
| Smoke habit^&^ | 12 (8.5) | 0 (0.0) | 0.440 |
| CKD^&^ | 11 (7.7) | 0 (0.0) | 0.300 |
| Cancer^&^ | 10 (7.0) | 0 (0.0) | 0.320 |
| COPD^&^ | 5 (3.5) | 3 (23.1) | 0.002 |
| Heart failure^&^ | 6 (4.2) | 0 (0.0) | 0.450 |
| **Symptoms during follow up** |  |  |  |
| Fever (>37,5ºC)^&^ |  |  | 0.570 |
| Cough^&^ | 57 (40.1) | 8 (61.5) | 0.130 |
| Dyspnoea^&^ | 38 (26.8) | 10 (76.9) | <0.001 |
| Myalgias^&^ | 17 (12.1) | 5 (38.5) | 0.009 |
| Asthenia^&^ | 34 (23.9) | 7 (53.8) | 0.019 |
| Headache^&^ | 15 (10.6) | 3 (23.1) | 0.180 |
| Chest pain^&^ | 21 (14.8) | 4 (30.8) | 0.130 |
| Dysgeusia^&^ | 2 (1.4) | 1 (7.7) | 0.120 |
| Anosmia^&^ | 2 (1.4) | 2 (15.4) | 0.003 |
| GI symptoms^&^ | 37 (26.1) | 4 (30.8) | 0.710 |
| Number of symptoms |  |  |  |
| 1- 3^&^ | 81 (57.0) | 6 (46.2) | 0.034 |
| ≥4^&^ | 34 (23.9) | 7 (53.8) |  |
| **Chest X-ray at diagnosis** |  |  |  |
| Bilateral pneumonia^&^ | 103 (72.5) | 7 (53.8) | 0.160 |
| **Blood test at diagnosis** |  |  |  |
| Lymphocytes (10E3/Âµ) ^$^ | 1200.0 (900.0, 1500.0) | 1200.0 (800.0, 1400.0) | 0.600 |
| D-dimer (μg/L) ^$^ | 439.5 (288.5, 930.0) | 336.5 (177.0, 839.0) | 0.320 |
| Ferritin (μg/L) ^$^ | 443.5 (200.0, 1215.0) | 242.0 (242.0, 242.0) | 0.550 |
| CRP (mg/L) ^$^ | 60.0 (22.6, 120.2) | 99.2 (38.5, 136.1) | 0.590 |
| **Chest- Xray follow-up (day)** |  |  | 0.750 |
| Normal X-ray^&^ | 90 (63.4) | 6 (46.2) | 0.160 |
| **Blood test follow-up (day)** |  |  | 0.120 |
| Lymphocytes (10E3/ÂµL)^#^ | 2453.7 (933.9) | 2177.8 (626.1) | 0.390 |
| D-dimer(μg/L) ^$^ | 465.0 (260.0, 845.0) | 460.0 (340.0, 880.0) | 0.740 |
| CRP (mg/L) ^$^ | 1.2 (0.3, 3.4) | 2.1 (1.1, 4.5) | 0.150 |
| Ferritin (μg/L) ^$^ | 152.0 (42.0, 330.0) | 306.0 (106.0, 512.5) | 0.260 |
| **Acute complications** |  |  |  |
| Hospital Admission^&^ | 112 (78.9) | 11 (84.6) | 0.620 |
| ICU admission^&^ | 2 (1.4) | 2 (15.4) | 0.002 |
| **Follow-up at practice** |  |  |  |
| Pneumonia onset (days) ^#^ | 8.0 (4.2) | 8.2 (3.4) | 0.840 |
| GP´s follow up until recovery^$^ | 51.5 (41.0, 75.0) | 103.0 (98.0, 119.0) | <0.001 |
| Phone calls (number) ^#^ | 11.6 (5.7) | 17.9 (10.9) | <0.001 |
| **Long COVID complications** |  |  |  |
| Thromboembolism^&^ | 4 (2.8) | 2 (15.4) | 0.025 |
| Readmission^&^ | 8 (5.6) | 1 (7.7) | 0.760 |
| Home Oxygen Therapy^&^ | 6 (4.2) | 2 (15.4) | 0.082 |

**Legend**. &: number (%), #: mean (standard deviation), $: median (interquartile range), BMI (body mass index), COPD (chronic obstructive pulmonary disease), GI (gastrointestinal), CRP (C reactive protein), ICU (intensive unit care).
